# Epicardial Adipose Tissue Reflects the Presence of Coronary Artery Disease: Comparison with Abdominal Visceral Adipose Tissue

**DOI:** 10.1155/2015/483982

**Published:** 2015-01-27

**Authors:** Masayoshi Oikawa, Takashi Owada, Hiroyuki Yamauchi, Tomofumi Misaka, Hirofumi Machii, Takayoshi Yamaki, Koichi Sugimoto, Hiroyuki Kunii, Kazuhiko Nakazato, Hitoshi Suzuki, Shu-ichi Saitoh, Yasuchika Takeishi

**Affiliations:** Department of Cardiology and Hematology, Fukushima Medical University, 1 Hikarigaoka, Fukushima 960-1295, Japan

## Abstract

Accumulation of visceral adipose tissue is associated with a risk of coronary artery disease (CAD). The aim of this study was to examine whether different types of adipose tissue depot may play differential roles in the progression of CAD. Consecutive 174 patients who underwent both computed tomography (CT) and echocardiography were analyzed. Cardiac and abdominal CT scans were performed to measure epicardial and abdominal visceral adipose tissue (EAT and abdominal VAT, resp.). Out of 174 patients, 109 and 113 patients, respectively, presented coronary calcification (CC) and coronary atheromatous plaque (CP). The EAT and abdominal VAT areas were larger in patients with CP compared to those without it. Interestingly, the EAT area was larger in patients with CC compared to those without CC, whereas no difference was observed in the abdominal VAT area between patients with CC and those without. Multivariable logistic regression analysis revealed that the presence of echocardiographic EAT was an independent predictor of CP and CC, but the abdominal VAT area was not. These results suggest that EAT and abdominal VAT may play differential pathological roles in CAD. Given the importance of CC and CP, we should consider the precise assessment of CAD when echocardiographic EAT is detected.

## 1. Introduction

Despite the development of preventive medicine, coronary artery disease (CAD) is still a major cause of death throughout the world [1]. With respect to the primary prevention of CAD, detecting the presence of coronary arteriosclerosis is important because early and strict management of coronary risk factors may prevent the progression of coronary plaque instability in those patients. However, detecting the early stage of coronary plaque is challenging because mild plaque burden normally presents no symptoms, even during exercise. Coronary computed tomography (CT) angiography offers great advantages in regard to describing coronary plaque morphology with high spatial resolution [2]. Not only is it advantageous for coronary artery visualization, but CT imaging can also be used to visualize peripheral structures of the heart. Recent studies revealed that epicardial adipose tissue (EAT) mediated coronary plaque formation [3, 4] and is associated with fatal and nonfatal coronary events [5]. Importantly, because EAT locates within the pericardial sac, the coronary artery could be directly influenced by inflammatory cytokines, which are released from peri-coronary EAT [6]. Abdominal visceral adipose tissue (VAT) is also associated with plaque morphology [7], and inflammatory cytokine secretion as well as increased reactive oxygen species generation may contribute to its progression [8]. Different types of adipose tissue depot may play differential roles in the progression of CAD; however, this issue is not yet fully understood.

In the present study, we examined whether EAT is important to detect coronary arteriosclerosis compared to abdominal VAT, and echocardiographic assessment of EAT is useful to detect coronary arteriosclerotic changes noninvasively.

## 2. Materials and Methods

### 2.1. Subjects

We enrolled 174 patients who underwent 64-slice CT angiogram and echocardiography for the diagnosis of CAD in this study. Patients who had a history of open heart surgery, coronary stent implantation, acute coronary syndrome, and poor image data were excluded. The study protocol was approved by the institutional ethics committee, and written informed consent was obtained from all study subjects.

### 2.2. CT Scan Protocol

CT examinations were performed using a 64-slice CT scanner (Aquilion 64, Toshiba Medical Systems Co., Ltd., Tokyo, Japan) with a collimation of 64 × 0.5 mm, pixel size of 0.39 × 0.39 mm, gantry rotation time of 350 msec, and tube voltage of 120 kV. Tube current was varied between 250 and 550 mA. Patients received 20 mg of metoprolol tartrate to reduce heart rate under 65 beats per minute unless they had already received or experienced a contraindication to *β*-blocker therapy. Nitroglycerin (0.3 mg) was administrated sublingually before CT scanning. A noncontrast scan was performed at the level of the umbilicus in order to assess the abdominal VAT area. Contrast media (80 mL) were injected for a contrast-enhanced scan. The raw data of the scan were reconstructed using algorithms optimized for retrograde ECG-gated multislice spiral reconstruction. The reconstructed image data were analyzed by a computer workstation (Ziostation2, Ziosoft Inc., Tokyo, Japan).

### 2.3. Evaluation of Fat Area and CAD

Abdominal adipose tissue area was divided into two parts, visceral fat and subcutaneous fat, based on the line of the peritoneal membrane. These adipose tissue areas were measured using an application software (Fat Measurement, Toshiba Medical Systems). The EAT area was measured at the level of the left main coronary artery. EAT was defined as any tissues presenting between −230 Hounsfield Unit (HU) and −30 HU enclosed by the visceral pericardium [9]. Coronary atheromatous plaque (CP) was defined as structures with less than 130 HU on coronary vessel walls. Coronary calcification (CC) was determined as structures with greater than 130 HU on vessel walls [10]. Coronary arteriosclerotic change was defined as any visible CP and/or CC based on CT findings.

### 2.4. Evaluation of Echocardiographic Epicardial Fat Thickness

Echocardiographic EAT (eEAT) was identified as a low-echoic area in the epicardial layers. A distinction between pericardial adipose tissue and EAT was made based on the systolic movement of EAT sliding along the inner side of the pericardium. EAT thickness was measured from the parasternal long-axis views on B-mode echocardiography, and the thickness was measured perpendicularly at end-systole [11]. We defined the presence of eEAT as more than a 1.5 mm thickness of EAT because of the difficulty of distinction between EAT and noisy signal.

### 2.5. Statistics

Data are expressed as median with interquartile ranges. The Mann-Whitney test was used for unequal distribution. Logistic regression analysis was performed on anthropometric and clinical variables to identify correlates of CC and CP. The improvement from adding eEAT to discrimination and net reclassification of risks was assessed by comparing the area under the curve (AUC) of receiver operator characteristics and the estimation of both integrated discrimination improvement (IDI) and net reclassification improvement (NRI) [12]. All tests were two-tailed and *P* values <0.05 were considered statistically significant. All statistical analyses were performed using R software packages version 3.1.2 (R Development Core Team).

## 3. Results

**Figure 1:**
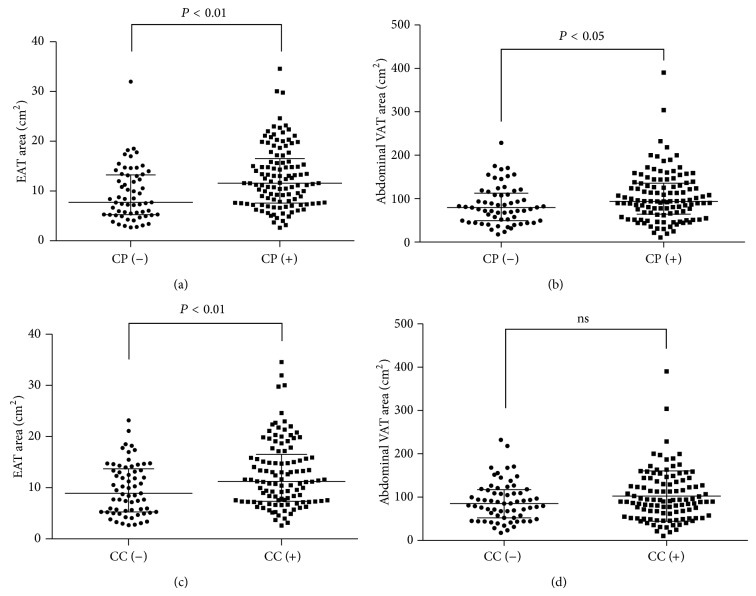
EAT and abdominal VAT areas in patients with CC or CP. CC (−) indicates non-CC. CC (+) indicates the presence of CC. CP (−) indicates non-CP. CP (+) indicates the presence of CP. Each point represents the adipose tissue area of the patient.

Patient characteristics are displayed in Table 1. Out of 174 subjects, 109 showed CC and 113 showed CP detected by CT angiography. Ten patients had only CC, 14 had only CP, 99 had both, and 51 had neither. Compared to the patients with normal coronary artery, those with either CC or CP had a higher age, hemoglobin A1c (HbA1c), and higher prevalence of hypertension. Regarding the VAT, both the EAT and abdominal VAT areas were larger in patients with either CC or CP. As expected, there was a good correlation between EAT area and abdominal VAT area (*R* = 0.710; *P* < 0.0001), and both areas of EAT and abdominal VAT were larger in patients with CP compared to those without CP (Figures 1(a) and 1(b)). With respect to CC, the EAT area was larger in CC patients; however, the abdominal VAT area was similar to those without CC (Figures 1(c) and 1(d)).

**Figure 2:**
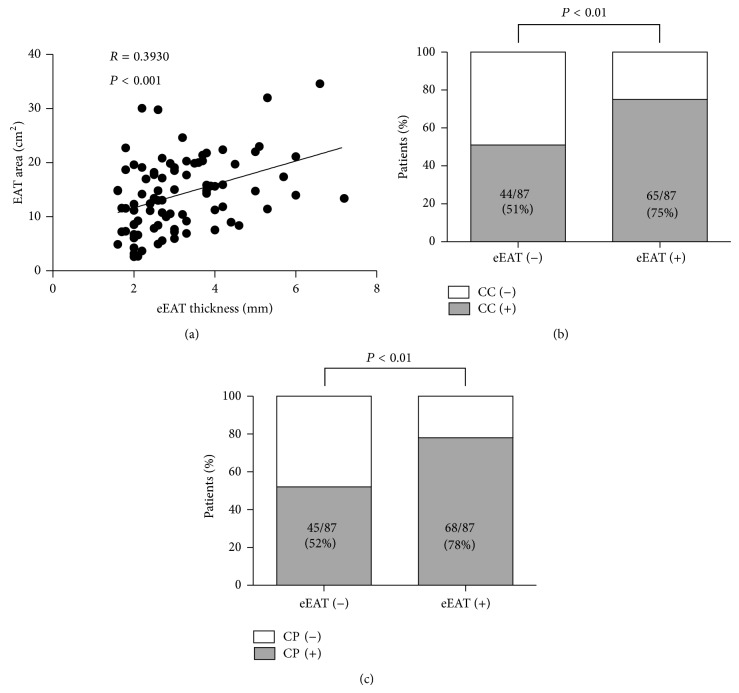
Association of eEAT and coronary arteriosclerotic changes. (a) Correlation between EAT areas assessed by CT and eEAT thickness assessed by echocardiogram. Each point represents the value of the patient. Subjects (*n* = 87) who showed more than 1.5 mm thickness of eEAT were used in this analysis. (b) Prevalence of CC between patients with and without the presence of eEAT. CC (−) indicates non-CC. CC (+) indicates the presence of CC. (c) Prevalence of CP between patients with and without the presence of eEAT. CP (−) indicates non-CP. CP (+) indicates the presence of CP.

Although CT is able to provide us with many informative findings with high-resolution images, the trade-off is radiation exposure. On the contrary, echocardiogram is usually used to obtain cardiac function and can be performed noninvasively and easily with no radiation exposure. Therefore, echocardiographic assessment of EAT would be a more desirable method. To analyze the impact of eEAT on arteriosclerosis, we compared eEAT data with CT findings. Out of 174, 87 patients had eEAT more than 1.5 mm thickness.  Figure 2(a) showed that there was significant correlation between eEAT thickness and EAT area by CT. As for EAT distribution in those without any eEAT, median and interquartile ranges of EAT area by CT were 8.0 cm^2^ [5.7 cm^2^–12.0 cm^2^]. Consistent with CT findings, the patients with eEAT showed a higher prevalence of CC and CP (Figures 2(b) and 2(c)). The diagnostic accuracy of presence of eEAT to detect CC or CP was as follows: sensitivity 0.596 and 0.601; specificity 0.662 and 0.689; positive predictive value 0.747 and 0.782; negative predictive value 0.494 and 0.483, respectively.

As shown in Table 2, univariable logistic regression analysis revealed that age, hypertension, HbA1c, the presence of eEAT, and abdominal VAT area were associated with CP. Multivariate logistic regression analysis indicated that age and presence of eEAT, but not abdominal VAT, were independent variables of CP. On the other hand, univariable logistic regression study revealed that age, hypertension, HbA1c, and the presence of eEAT were associated with CC. Among these factors, age, HbA1C, and the presence of eEAT were independent variables of CC (Table 3). Value of AUC increased when eEAT was added to the model, although it did not reach statistical significance. The difference in AUC was 1.8% (*P* = 0.28) for CC and 1.9% (*P* = 0.36) for CP. To assess the importance of adding eEAT for predicting CC and CP, NRI and IDI were calculated using the variables with or without eEAT. The values of NRI and IDI for detecting CC increased significantly when eEAT was added to the model: 0.5806 (95% CI, 0.2341–0.9272, *P* = 0.001) for NRI and 0.0264 (95% CI, −0.0036–0.0564, *P* = 0.08) for IDI. As for CP, the value of NRI was 0.6503 (95% CI, 0.2987–1.0019, *P* = 0.0003) and IDI was 0.0327 (95% CI, 0.0016–0.0638, *P* = 0.04).

## 4. Discussion

The summary of this study is as follows. First, accumulation of visceral adipose tissue, both EAT and abdominal VAT, was associated with arteriosclerotic changes of the coronary artery. Second, EAT was associated with CC, whereas abdominal VAT was not. Third, the presence of eEAT was independently associated with increased risk of CP and CC.

### 4.1. Association of EAT and Abdominal VAT with CAD

EAT shares an embryological origin, splanchnopleuric mesoderm, with abdominal VAT [13, 14]. EAT expresses a high amount of inflammatory mediators such as interleukin-1*β*, interleukin-6, monocyte chemotactic protein-1, and tumor necrosis factor-*α* (TNF-*α*) [15]. A recent report showed that macrophage infiltration and the ratio of M1/M2 macrophages in EAT were positively enhanced with the severity of CAD [16]. Likewise, abdominal VAT also enhanced TNF-*α* production, resulting in a reduction of nitric oxide availability by promoting superoxide generation [17], and the abdominal VAT area was associated with the presence of noncalcified coronary plaque with positive remodeling [7]. Though both types of VAT share the same origin and similar inflammatory effects, the impact of adipose tissues on arteriosclerosis might be different in EAT to that in abdominal VAT. A community-based cohort study revealed that abdominal VAT has a strong correlation of metabolic risk factors. Meanwhile, EAT is associated with CC [18]. Considering the fact that abdominal VAT produces metabolites, inflammatory cytokinesis, and hormones, which were distributed to whole body through systemic circulation [19], it could contribute to the promotion of not only coronary plaque formation but systemic metabolic disease as well. On the other hand, EAT is much smaller than abdominal VAT, but it directly adheres to coronary arteries to induce inflammatory changes on the vessel wall efficiently. de Vos and colleagues showed that EAT thickness around the coronary artery was strongly correlated with CC [20]. Consistent with this concept, the present study showed that the area of EAT was larger in patients with CC compared to those without it, but the area of abdominal VAT was not. CC is considered as an important marker for cardiovascular risk assessment, and calcium score is used to represent the severity of CC [21]. Although high calcium score increased the cumulative incidence of coronary events proportionally [22], several studies revealed that the presence of CC, even a small amount of calcification, increased the cardiac risk compared to the patients with zero-calcium score [23, 24]. As for CP, 5-year follow-up of the DEFER study showed that the risk of myocardial infarction or cardiac death in nonischemic coronary stenosis was less than 1% per year [25]. From their findings, it seems that the presence of mild plaque is not important in clinical situation. However, the majority of them were treated with statin therapy in the DEFER study, and Falk et al. showed that plaque rupture occurred in the lesion with mild plaque burden assessed by serial angiographic examination [26]. In addition, a systematic review shows that nonobstructive CAD assessed by CT significantly increases odds of cardiovascular events compared to absence of CAD [27]. Therefore, we think the detection of coronary plaque and/or calcification, even if mild accumulation, is important because we can choose the strategy to prevent cardiac event by statin therapy, modifying lifestyle habit, and so forth. In the current study, we demonstrated that the presence of eEAT was an independent predictor of the presence of CC and CP. By adding the eEAT, the *P* value of IDI for CC was not less than 0.05, but NRI reached statistical significance, and eEAT significantly improved the prediction of CP. These results supported the importance of eEAT to detect the presence of coronary arteriosclerotic changes. Given the cardiac risk in the future, assessment of eEAT could be a useful screening method to detect asymptomatic CAD patients who need proper medical management.

We also have to take into account that approximately 50% patients without eEAT also have arteriosclerotic changes. Because many factors are associated with plaque formation, we should not exclude the risk of coronary arteriosclerosis in patients without eEAT. However, if eEAT is detected, precise assessment of arteriosclerosis should be employed.

### 4.2. Limitations

First, we conducted this study using a relatively small number of patients and excluded lesions with inadequate image quality from analysis. Second, the patients who underwent CT analysis might be different from normal subjects, as they have symptoms or clinical features to be suspected of CAD. This raises the possibility that they were likely to be more exposed to an arteriosclerotic risk factor than the normal population. Third, we cannot assess the relationship between the severity of CC and EAT in this study because a measurement of calcium score was not employed in the study protocol in our facilities. Fourth, echocardiographic analysis has the advantage of noninvasiveness, but quantification of EAT is difficult. Recent development of three-dimensional echocardiographic techniques may provide us with a new method to quantitate EAT in the future.

## 5. Conclusions

Our study showed that the presence of eEAT, but not abdominal VAT, was associated with the presence of CC and CP, suggesting that EAT and abdominal VAT may play differential pathological roles in CAD. Given the importance of CC and CP, we should consider the precise assessment of CAD when eEAT is detected.

## Figures and Tables

**Table 1:** Patient characteristics.

	Normal (*n* = 51)	Calcification (*n* = 109)	Atheromatous plaque (*n* = 113)
Age (years)	58 [44–69]	72 [67–77]^**^	71 [65–77]^**^
BMI (kg/m^2^)	23.4 [21.5–26.5]	23.7 [21.8–26.0]	23.9 [22.0–26.6]
Male (*n*, %)	29, 57%	68, 63%	73, 65%
Hypertension (*n*, %)	29, 47%	80, 73%^**^	82, 73%^**^
LDL-C (mg/dL)	109 [97–131]	99 [85–127]	100 [85–126]
HbA1c (%)	5.7 [5.3–6.1]	6.0 [5.7–6.8]^**^	6.0 [5.7–6.8]^**^
Smoking history (*n*, %)	17, 33%	50, 46%	53, 47%
EAT area (cm^2^)	8.4 [5.1–13.1]	11.3 [7.4–16.5]^**^	11.6 [7.6–16.5]^**^
Abdominal VAT area (cm^2^)	79.2 [49.2–109.3]	91.1 [59.8–135.7]^*^	94.1 [64.6–135.7]^*^
Subcutaneous fat area (cm^2^)	167.5 [98.1–222.2]	127.6 [94.4–178.6]	128.7 [102.2–182.9]
Presence of eEAT (*n*, %)	18, 35%	65, 60%^**^	68, 60%^**^

Values are expressed as median with interquartile ranges.

BMI, body mass index; LDL-C, low-density lipoprotein cholesterol; HbA1c, hemoglobin A1c; EAT, epicardial adipose tissue; VAT, visceral adipose tissue; eEAT, echocardiographic epicardial adipose tissue; ^*^ *P* < 0.05, ^**^ *P* < 0.01 versus normal group.

**Table 2:** Association with the presence of coronary atheromatous plaque.

	Univariate		Multivariate	
	OR (95% CI)	*P* value	OR (95% CI)	*P* value
Age, per 5 years	1.522 (1.301–1.778)	<0.001	1.657 (1.326–2.064)	<0.001
BMI, per 1 kg/m^2^ increase	1.025 (0.945–1.112)	0.549		
Hypertension	2.560 (1.336–4.904)	0.005	1.450 (0.528–3.986)	0.471
LDL-C, per 1 mg/dL increase	0.992 (0.981–1.004)	0.195		
HbA1c, per 1% increase	1.644 (1.055–2.562)	0.028	1.501 (0.952–2.368)	0.080
Smoking history	1.589 (0.836–3.023)	0.158		
Presence of eEAT	3.340 (1.727–6.462)	<0.001	2.844 (1.100–7.351)	0.031
Abdominal VAT area, per 10 cm^2^ increase	1.083 (1.010–1.161)	0.028	1.073 (0.970–1.172)	0.165

OR, odds ratio; CI, confidence interval; BMI, body mass index; LDL-C, low-density lipoprotein cholesterol; HbA1c, hemoglobin A1c; eEAT, echocardiographic epicardial adipose tissue; VAT, visceral adipose tissue.

**Table 3:** Association with the presence of coronary calcification.

	Univariate		Multivariate	
	OR (95% CI)	*P* value	OR (95% CI)	*P* value
Age, per 5 years	1.707 (1.429–2.038)	<0.001	1.649 (1.326–2.047)	<0.001
BMI, per 1 kg/m^2^ increase	1.007 (0.930–1.089)	0.871		
Hypertension	2.675 (1.402–5.102)	0.003	2.203 (0.831–5.838)	0.112
LDL-C, per 1 mg/dL increase	0.994 (0.983–1.006)	0.334		
HbA1c, per 1% increase	1.770 (1.134–2.762)	0.012	1.721 (1.076–2.753)	0.023
Smoking history	1.525 (0.811–2.869)	0.190		
Presence of eEAT	2.887 (1.522–5.479)	0.001	2.653 (1.064–6.618)	0.036
Abdominal VAT area, per 10 cm^2^ increase	1.051 (0.990–1.116)	0.134		

All abbreviations as in Table 2.
